# Preoperative Serum IL-12p40 Is a Potential Predictor of Kasai Portoenterostomy Outcome in Infants with Biliary Atresia

**DOI:** 10.1155/2017/9089068

**Published:** 2017-05-15

**Authors:** Shaimaa Samy Goda, Mohamed Ahmed Khedr, Soha Zaki Elshenawy, Tarek Mohamed Ibrahim, Hanaa Ahmed El-Araby, Mostafa Mohamed Sira

**Affiliations:** ^1^Department of Pediatric Hepatology, Gastroenterology and Nutrition, National Liver Institute, Menoufia University, Shebin El-Koom, Menoufia 32511, Egypt; ^2^Department of Biochemistry, National Liver Institute, Menoufia University, Shebin El-Koom, Menoufia 32511, Egypt; ^3^Department of Hepatobiliary Surgery, National Liver Institute, Menoufia University, Shebin El-Koom, Menoufia 32511, Egypt

## Abstract

The standard-of-care treatment for biliary atresia (BA) is surgical restoration of bile flow by Kasai portoenterostomy. We aimed to study serum interleukin- (IL-) 12p40, a natural antagonist for the proinflammatory IL-12p70, and its relation to surgical outcomes of BA. The study included 75 infants with neonatal cholestasis: BA group (*n* = 25), non-BA cholestasis group (*n* = 30), and neglected BA group (*n* = 20), in addition to thirty healthy neonates serving as controls. IL-12p40 was measured by ELISA in all individuals and a second assessment was performed 3 months postoperatively in the BA group. The surgical outcomes were classified as successful (bilirubin ≤ 2 mg/dl) or failed (bilirubin > 2 mg/dl). IL-12p40 was higher in BA compared to that in the non-BA and control groups (*P* values were 0.036 and <0.0001, resp.) but comparable to that in the neglected BA group. Preoperative IL-12p40 levels in BA patients were significantly higher in successful Kasai compared with failed Kasai and a cutoff level of 547.47 pg/ml could predict the successful outcome with 87.5% sensitivity and 82.4% specificity. Three-month postoperative IL-12p40 tended to decrease in both the successful and failed groups. In conclusion, preoperative serum IL-12p40 is a potential predictor of Kasai outcome. Serial postoperative measurements may anticipate the failure of an initially successful operation, hence the need for liver transplantation.

## 1. Introduction

Biliary atresia (BA) is a neonatal disease that is characterized by progressive fibroinflammatory cholangiopathy usually manifesting in the first month of life. It causes severe cholestasis, rapidly progressing biliary cirrhosis, and death in the first years of life, if left untreated [1]. There is a little information on the etiopathogenesis of BA, and this hardens prevention strategies and therapies designed to stop progression of the fibroinflammatory process of the bile ducts. Several hypotheses have been explored as viral infection which is considered as a triggering factor, autoimmune response against antigens from the biliary epithelia, and genetic susceptibility [2].

The treatment of BA is surgical during the first months of life. A hepatoportoenterostomy “Kasai” operation should be performed to restore the biliary flow to the intestine and to lessen further damage to the liver. If this fails, or the disease progresses to biliary cirrhosis and life-threatening complications, liver transplantation is then necessary, for which BA represents the most frequent indication in the pediatric age group. Of importance, the earlier the Kasai is performed, the later the liver transplantation is usually needed [3].

The pathogenesis of BA is not only associated with mechanical obstruction but also associated with an inflammatory process involving the biliary tree. A progressive inflammatory process characterized by increased production of proinflammatory, T-helper 1 (Th1), Th2, and macrophage cytokines has been demonstrated in children with BA [4].

The roles of various inflammatory factors, including natural killer (NK) cells, T lymphocytes, the proinflammatory interleukin- (IL-) 12p70 and its receptor's competing ligand IL-12p40, Th1 cytokines (IL-2 and interferon-gamma), Th2 cytokine (IL-10), and macrophage markers (tumor necrosis factor-alpha and transforming growth factor-beta), have been reported in BA patients [5]. We have previously studied different proinflammatory molecules such as intercellular adhesion molecule-1 [6], CD56 [7], and P-selectin [8] and different proinflammatory cytokines such as IL-2 and IL-8 [9] in BA and described their relation to the disease process.

As various inflammatory processes contribute to the pathogenesis of BA, it is speculated that different clinical outcomes of BA may be attributed to the severity of inflammation. The serum cytokine patterns before and after Kasai operation in patients with BA may help to identify potential biomarkers that can predict short- and long-term outcomes of the Kasai operation [10]. IL-12p40 is one of the important cytokines that is secreted by activated macrophages, dendritic cells, neutrophils, and microglia. It was found that IL-12p40 competitively inhibits IL-12p70 receptors, suppressing the proinflammatory responses [11].

The aim of the current study is to investigate serum IL-12p40 and its relation to the outcome of Kasai operation in BA patients.

## 2. Patients and Methods

### 2.1. Study Population

This prospective study included 75 infants with neonatal cholestasis in whom liver biopsy was indicated for etiological diagnosis. They were divided into three groups: group 1, the BA group (*n* = 25) all of whom underwent Kasai hepatoportoenterostomy and were followed up for 3 months postoperatively; group 2, the non-BA cholestasis group (*n* = 30 with cholestasis due to causes other than BA); and group 3, the neglected BA group (*n* = 20) being those BA patients with delayed diagnosis and lost chance for the corrective surgery. The BA group was further divided after 3 months postoperatively according to the total bilirubin level into successful outcome (total bilirubin < 2 mg/dl) and failed outcome (total bilirubin ≥ 2 mg/dl) [12]. All patients were recruited from the Department of Pediatric Hepatology, Gastroenterology and Nutrition, National Liver Institute, Menoufia University, Egypt. A fourth group of healthy neonates (*n* = 30) served as healthy controls. A written informed consent was obtained from the parents or the legal guardians of the patients and controls before enrollment in the study. The study was approved by the Research Ethics Committee of the National Liver Institute, Menoufia University, Egypt.

### 2.2. Etiological Diagnosis

After full history taking, thorough clinical examination, and routine investigations, the patients in each group were allocated as BA and non-BA by our newly developed BA diagnostic score [13]. Diagnosis of BA was confirmed by intraoperative cholangiography and/or laparotomy prior to surgery. Routine investigations included total and direct bilirubin, total serum proteins, albumin, alanine transaminase, aspartate transaminase, alkaline phosphatase, gamma-glutamyl transpeptidase, prothrombin time (PT), complete blood count, viral antibodies (immunoglobulin [Ig]M and IgG for rubella, cytomegalovirus, herpes simplex virus types 1 and 2, and hepatitis B virus core), toxoplasma antibodies (both IgM and IgG), hepatitis B surface antigen, ultrasonography (US) and Doppler US, and liver biopsy. Follow-up in the non-BA group, together with a set of specific laboratory tests according to the expected etiology, the diagnosis of BA was ruled out in the patients of this group. Their diagnoses were progressive familial intrahepatic cholestasis (*n* = 17), idiopathic neonatal hepatitis (*n* = 3), paucity of intrahepatic bile ducts (*n* = 5), cytomegalovirus hepatitis (*n* = 2), glycogen storage disease type 4 (*n* = 2), and galactosemia (*n* = 1).

### 2.3. Liver Biopsy

US-guided liver biopsy was performed for all patients using a Tru-Cut needle (GTA, Quistello, MN, Italy). A core of a liver tissue containing at least 5 portal tracts was considered sufficient. Biopsy specimens were fixed in formalin and embedded in paraffin. 5 μm thick sections were cut, mounted on glass slide, and stained with hematoxylin and eosin to evaluate pathological changes, with Masson-Trichrome that stains collagen fibers to assess fibrosis, and with Perls' Prussian blue stain which reveals iron deposits. Portal fibrosis and inflammatory activity were assessed using semiquantitative histopathological scores as described in Russo et al. [14].

### 2.4. Serum IL-12p40

Serum samples were collected from all the patients and controls in addition to follow-up samples that were collected from the BA group (*n* = 25) 3 months after Kasai operation. Samples were stored in aliquots at −80°C till the time of the assay. Quantitative assessments of human serum IL-12p40 levels were tested by enzyme-linked immunosorbent assay (ELISA) kit (from Boster Biological Technology Co. Ltd., CA, USA; EK0423) according to the manufacturer's instructions. The detection limit of the assay is up to 2000 pg/ml.

### 2.5. Statistical Analysis

Descriptive results were expressed as mean ± standard deviation (mean ± SD) or number and percentage. For quantitative data, significance between two groups was tested by the Mann–Whitney *U* test and significance between more than two groups was tested by the Kruskal-Wallis test. A paired *t*-test was used to assess the difference in serum cytokine levels before and after Kasai operation. For qualitative and categorical data, significance was tested by chi-square test or Fisher's exact test. Correlation was tested by Spearman's test. The diagnostic value of serum IL-12p40 was assessed by calculating the area under the receiver-operating characteristic (ROC) curve. The cutoff value for optimal clinical performance was determined from the ROC curves. The diagnostic performance was measured as sensitivity, specificity, positive predictive value (PPV), and negative predictive value (NPV) and expressed as percentages. Significance was set to *P* < 0.05. Statistical analysis was performed using SPSS software version 13 (SPSS Inc., Chicago, IL, USA).

## 3. Results

### 3.1. Study Population's Characteristics

The current study included 105 infants divided into the BA group (*n* = 25) with ages ranged from 40 to 80 days, the neglected BA group (*n* = 20) with ages ranged from 84 to 360 days, the non-BA group (*n* = 30) with ages ranged from 30 to 90 days, and the control group (*n* = 30) with ages ranged from 30 to 80 days. All groups were sex matched (*P* = 0.75). Apart from the neglected BA group, all the other groups were age matched (*P* = 0.124). The occurrence of clay-colored stool and serum levels of gamma-glutamyl transpeptidase were significantly higher in the BA and neglected BA groups than in the non-BA group (*P* < 0.0001 for both). On the other hand, occurrence of ascites was significantly higher in the neglected BA group than in the other studied groups (*P* = 0.018). PT was significantly higher in the neglected BA group and the non-BA group than in the BA group (*P* = 0.017). Other baseline demographic, clinical, and laboratory parameters were comparable in both groups (Table 1).

### 3.2. Histopathological Findings

The occurrence of higher grades of portal fibrosis was significantly higher in the BA group (60% with focal porto-portal bridging) and the neglected BA group (50% with marked bridging and 10% with cirrhosis) than in the non-BA group (20% with focal porto-portal bridging and 6.7% with marked bridging) (*P* < 0.0001). Portal cellular infiltrate was comparable in the BA and non-BA groups (*P* = 0.106), while moderate/severe portal cellular infiltrate was higher (30%) in the neglected BA group (Table 2).

### 3.3. TORCH Serological Markers in the Studied Patients

The occurrence of toxoplasma IgG and HSV-1 IgG was significantly higher in the BA and non-BA groups than in the neglected BA group (*P* = 0.026 and 0.008, resp.), and cytomegalovirus IgM was significantly higher in the non-BA group than in the BA and neglected BA groups (*P* = 0.048) while there was no significant statistical difference between the studied groups regarding the other TORCH serological markers (Table 3).

### 3.4. Comparison of Preoperative Serum IL-12p40 in the Studied Groups

**Figure 1:**
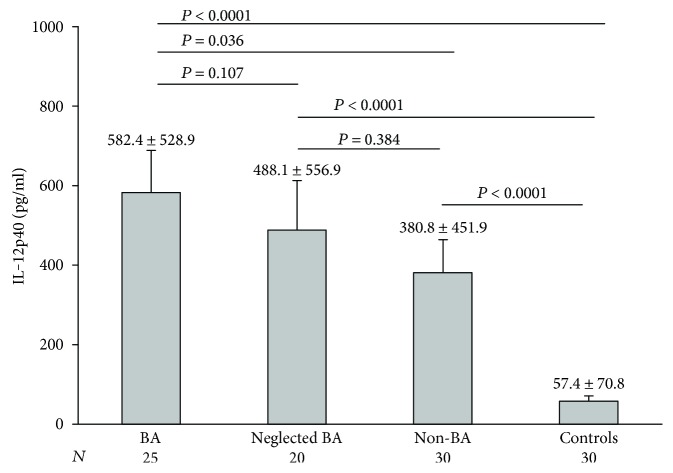
Preoperative serum IL-12p40 in the studied groups. The columns represent the mean, while the error bars represent the standard deviation. The horizontal bar represents the significance between the designated groups.

Preoperative serum levels of IL-12p40 were significantly higher in the BA group than in the non-BA group and the control group while there was no significant statistical difference between the BA and neglected BA groups. In addition, the levels were significantly higher in the neglected BA group compared with those in the controls but not with the non-BA group. Lastly, the levels in the non-BA group were significantly higher than those in the controls (Figure 1).

### 3.5. Comparison of Basic Characteristics and Serum IL-12p40 between BA Patients with Successful and Those with Failed Kasai Operation

**Figure 2:**
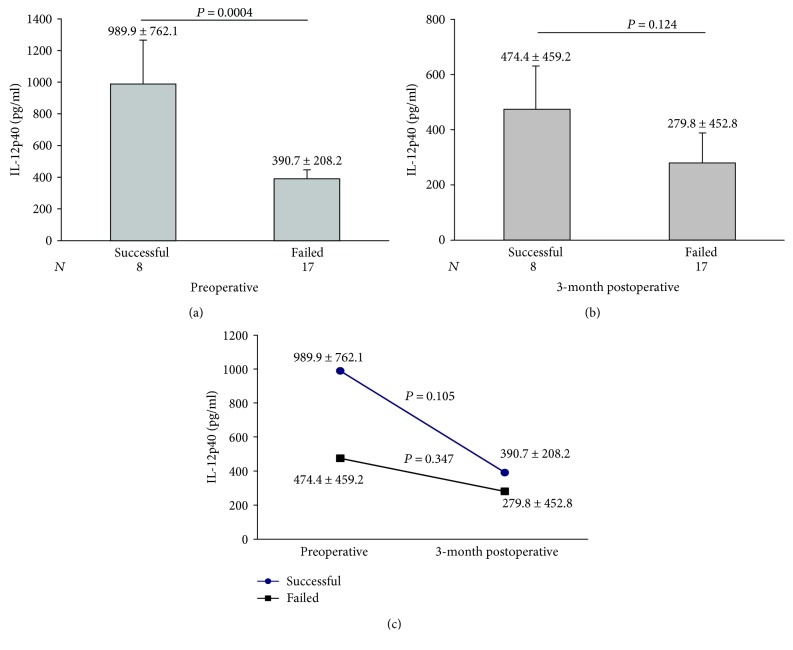
Comparison of serum IL-12p40 between BA patients with successful and those with failed Kasai operation. (a) Preoperative and (b) 3-month postoperative (c) paired analysis of preoperative and the 3-month postoperative IL-12p40 levels in patients with successful Kasai (upper slope) and those with failed Kasai (lower slope).

There was no significant statistical difference between BA patients with successful and those with failed Kasai regarding the basic demographic, clinical, laboratory, and histopathological parameters (*data not shown*), while the preoperative serum levels of IL-12p40 were significantly higher in those with successful compared to those with failed Kasai (Figure 2(a)). The 3-month postoperative levels of IL-12p40 did not differ significantly between both groups (Figure 2(b)). Serum levels of IL-12p40 tended to decrease 3 months postoperatively in both groups with the levels in the successful group approaching the levels in the failed group, though this decrease was not statistically significant in both groups (*P* = 0.105 and 0.347, resp.) (Figure 2(c))

### 3.6. Clinical Performance of Preoperative Serum IL-12p40 in Predicting Successful Kasai

**Figure 3:**
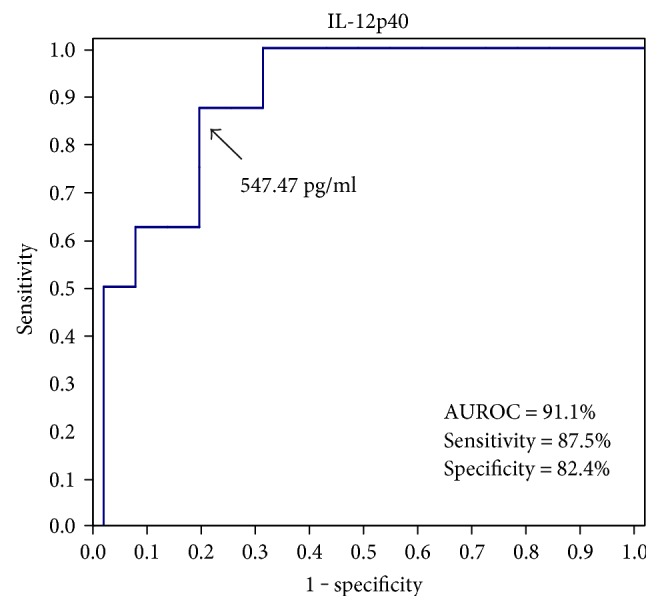
Performance of preoperative serum IL-12p40 in discriminating between BA patients with successful and those with failed Kasai. AUROC: area under receiver-operating characteristic.

Preoperative serum IL-12p40 at a cutoff value of 547.47 pg/ml could predict BA patients with successful Kasai by 87.5% sensitivity, 82.4% specificity, 70% PPV, and 93.3% NPV (Figure 3).

### 3.7. Correlation of IL-2p40 with the Studied Parameters in All Individuals

Serum levels of IL-12p40 did not correlate significantly with any of the studied parameters except for PT which had a marginal significance (*P* = 0.053) (Table 4).

## 4. Discussion

In the current study, 8 out of 25 (32%) patients had successful Kasai, while the other 17 patients had failed Kasai. Several factors have been shown to affect the outcome of Kasai portoenterostomy. The outcome is worse in the embryonic form compared to that in the perinatal form of BA [15]. In addition, type 3 BA is associated with a poor outcome [16]. Our center experience revealed that type 3 atresia comprises about 90% of our patients [17]. This may explain the low success rate in our study. In hand with our results, Sangkhathat et al. [18] reported a success rate of 33.4%. Similarly, Wildhaber et al. [19] and Chung et al. [20] reported success rates of 38% and 36%, respectively. Yet, some other studies reported slightly higher success rates ranging from 43% to 50% [21–23].

The role of inflammatory cytokines has been extensively studied in BA [4, 9, 24, 25]. IL-12 [26] and IL-23 [27] were found to be significantly higher in BA than in controls. IL-12p40 is a subunit shared by both IL-12 (with p35 subunit) and IL-23 (with p19 subunit) [28, 29]. IL-12p40 and IL-12p35 are the products of two separate genes that are differentially controlled [30]. Association of IL-12p35 and IL-12p40 subunits forms the bioactive heterodimer of 70–75 kDa (IL-12p70 or alternatively IL-12p75). Dendritic cells, monocytes, and macrophages secrete a 10–1000-fold excess of free p40 compared to p35 [31]. This excess amount of IL-12p40 (monomer) may also dimerize to from IL-12p80 (homodimer). Both the monomer and the homodimer have been shown to be natural antagonists to IL-12p70 [32, 33] and IL-23 [34] by competing for binding to IL-12R-beta1. In other words, the proinflammatory IL-12p70 and IL-23 are antagonized by their own IL-12p40 subunit [11].

In the current study, we found that preoperative serum levels of IL-12p40 were significantly higher in those with successful Kasai compared to those with failed Kasai (*P* = 0.0004). It seems that IL-12p40, as an inhibitor to both IL-12p70 and IL-23, carries a better prognosis to Kasai operation. IL-12p40 at a cutoff level of 547.47 pg/ml could predict a successful outcome with 87.5% sensitivity, 82.4% specificity, and AUROC of 91.1%. Wu et al. [35] reported similar results where those with successful Kasai had a median IL-12p40 level of 65.71 pg/ml compared to 30.09 pg/ml in those with failed surgery and a cutoff level of 33 pg/ml could discriminate those with favorable outcome at 3 months postoperatively with 89.5% sensitivity, 71.4% specificity, and AUROC of 81%.

In the 3-month postoperative follow-up, IL-12p40 levels decreased when compared with the preoperative levels in both the successful and the failed Kasai groups, yet the levels were still higher in those with successful outcome (390.7 ± versus 279.8 pg/ml). Similarly, Wu et al. [35] reported the decrease of the median values from 54.74 pg/ml in preoperative levels compared to 44.41 pg/ml in the 3-month postoperative levels (*P* = 0.12).

It is plausible to suggest that IL-12p40 secretion is stimulated early in the course of the disease and the level declines as time passes. This is also noticed in the lower levels found in the neglected BA group with longer disease duration (141.05 ± 60.69 days) compared to the BA group (56.56 ± 11.55 days). The fibroinflammatory process in BA is an ongoing one, leading to progressive scarring of the liver and severe fibrosis ending in cirrhosis even after successful surgery and the surgical reconstitution of biliary flow did not stop the decrease in IL-12p40 levels. This is probably due to the persistent release of proinflammatory cytokines by the primed immune cells in the portal tracts around the bile duct vicinity [36].

This finding enforces a question to be asked. What will happen to IL-12p40 beyond the 3-month follow-up period and whether the Kasai outcome may change? The answer to this question necessitates a longitudinal study with serial follow-up measurements of IL-12p40. If the levels decrease below the cutoff level, failure of operation is then anticipated and liver transplantation can be thought of.

Infections are a known stimulus to IL-12 secretion [31]. In the current study, the occurrence of serum markers of TORCH infection was in favor of the non-BA group. Yet, serum levels of IL-12p40 were significantly higher in the BA group. It is possible to conclude that TORCH infection was not a determining factor for the significantly increased secretion of IL-12p40 in the BA group when compared with the non-BA group.

In the current study, we could not find a significant correlation between IL-12p40 serum levels and the studied preoperative parameters, including the stage of portal fibrosis and inflammatory activity in liver biopsies. The negative correlation coefficient for both fibrosis and activity denotes a negative association, yet it was insignificant. A similar finding was reported by Wu et al. [35].

The relation between IL-12p40 and fibrosis is controversial. While some studies describe IL-12p40 as antifibrotic [37], other studies reported a profibrotic activity [38]. Sometimes the terms IL-12p40 and IL-12 are used interchangeably, and measurements of the p40 chain are often interpreted as measurements of the intact p70 heterodimer, such interchangeable usage may be incorrect and misleading.

It is important to emphasize that IL-12p40 is not the only driving force for the fibroinflammatory process in BA. Nonetheless, it is one of the multiple cytokines and mediators that are acting in the disease process. Additional studies are required to confirm the precise relationship among IL-12p40, IL-12p70, and other related cytokines in the biliary tree. IL-12p40 knockout mice manifest more severe portal inflammation and bile duct damage, including signs of portal hypertension and liver fibrosis [39].

The stimulation of IL-12p40 gene is independent of IL-12p35 gene and hence independent IL-12p70 production. The selective stimulation of the inhibitory IL-12p40 can be thought of as an anti-inflammatory and a natural immunosuppressive adjuvant treatment in infants with BA, specially postoperative to improve the outcome. Prostaglandin E2 was reported to selectively induce the production of IL-12p40 without the induction of the bioactive IL-12p70 [40]. Alternatively, the use of an inhibitor that selectively interferes with the effect of the complete IL-12p70 and IL-23 molecules but not with the inhibitory IL-12p40 subunit would be an ideal approach to improve Kasai outcome. Clarke et al. [41] reported an antibody with such described activity.

In conclusion, higher preoperative serum IL-12p40 can predict successful Kasai outcome with acceptable performance. Postoperative follow-up of IL-12p40 serum levels would help to anticipate failure of initially successful surgery. The selective induction of IL-12p40 in such patients may improve the surgical outcome. Longer follow-up is needed to evaluate the association of IL-12p40 with the long term outcome of Kasai operation. Future studies using selective IL-12p40 inducers or antibodies selectively blocking IL-12p70 but not p40 are worthy.

## Figures and Tables

**Table 1:** Demographic, clinical, and laboratory characteristics of the studied patients.

Characteristics	BA (*n* = 25)	Neglected BA (*n* = 20)	Non-BA (*n* = 30)	*P* value
Age at liver biopsy (days)	56.56 ± 11.55	141.05 ± 60.69	57.60 ± 18.90	<0.0001
Male, *n* (%)	14 (56)	9 (45)	16 (53.3)	0.75
Clay stool	25 (100)	20 (100)	10 (33.3)	<0.0001
Hepatomegaly (US)	25 (100)	19 (95)	38 (93.3)	0.438
Splenomegaly (US)	16 (64)	16 (80)	16 (53.3)	0.157
Ascites (US)	0 (0.0)	4 (20)	1 (3.3)	0.018
Total bilirubin (mg/dl)	11.32 ± 4.1	12.12 ± 4.23	10.77 ± 4.75	0.424
Direct bilirubin (mg/dl)	7.74 ± 3.48	9.08 ± 3.92	7.72 ± 4.22	0.29
Alanine transaminase (U/l)	141.86 ± 129.95	149.2 ± 114.16	168.37 ± 112.91	0.465
Aspartate transaminase (U/l)	200.04 ± 129.69	274.15 ± 197.89	347.0 ± 258.97	0.058
Albumin (g/dl)	3.69 ± 0.46	3.23 ± 0.62	3.5 ± 0.69	0.11
Alkaline phosphatase (U/l)	568.68 ± 300.02	738.0 ± 349.57	725.37 ± 359.18	0.092
Gamma-glutamyl transpeptidase (U/l)	1066.28 ± 762.45	832.2 ± 634.82	223.53 ± 265.61	<0.0001
Prothrombin time (sec)	12.42 ± 1.65	14.36 ± 2.46	14.45 ± 6.73	0.017

BA: biliary atresia.

**Table 2:** Histopathological characteristics of the studied patients.

Characteristics	BA (*n* = 25)	Non-BA (*n* = 30)	Neglected BA (*n* = 20)	*P* value
Grade of liver fibrosis				
Absent or fibrous expansion of some portal tracts	3 (12)	11 (36.7)	0 (0.0)	<0.0001
Fibrous expansion of most portal tracts	7 (28)	11 (36.7)	4 (20)	
Focal porto-portal bridging	15 (60)	6 (20)	4 (20)	
Marked bridging	0 (0.0)	2 (6.7)	10 (50)	
Cirrhosis	0 (0.0)	0 (0.0)	2 (10)	
Portal cellular infiltrate				
No/minimal	11 (44)	11 (36.7)	9 (45)	0.106
Mild	11 (44)	17 (56.7)	5 (25)	
Moderate/severe	3 (12)	2 (6.7)	6 (30)	

BA: biliary atresia.

**Table 3:** Toxoplasma and viral infections in the studied groups.

Characteristics	BA (*n* = 25)	Neglected BA (*n* = 20)	Non-BA (*n* = 30)	*P* value
Rubella IgM	0 (0.0)	0 (0.0)	0 (0.0)	NA
Rubella IgG	13 (52)	7 (35)	17 (56.7)	0.307
Toxoplasma IgM	0 (0.0)	0 (0.0)	0 (0.0)	NA
Toxoplasma IgG	15 (60)	4 (20)	13 (43.3)	0.026
Cytomegalovirus IgM	1 (4)	1 (5)	7 (23.3)	0.048
Cytomegalovirus IgG	21 (84)	16 (80)	26 (86.7)	0.82
Herpes simplex virus-1 IgM	2 (8)	0 (0.0)	0 (0.0)	0.128
Herpes simplex virus-1 IgG	14 (56)	4 (20)	19 (63.3)	0.008
Herpes simplex virus-2 IgM	0 (0.0)	0 (0.0)	0 (0.0)	NA
Herpes simplex virus-2 IgG	5 (20)	6 (30)	3 (10)	0.201

BA: biliary atresia; NA: not applicable.

**Table 4:** Correlation of IL-2p40 with age, laboratory, and histopathological parameters of the studied patients.

Parameters	IL-12p40	
	*r*	*P* value
Age (days)	0.075	0.629
Total bilirubin (mg/dl)	0.198	0.089
Direct bilirubin (mg/dl)	0.114	0.33
Albumin (g/dl)	−0.0285	0.469
Alanine aminotransferase (U/l)	−0.02	0.865
Aspartate aminotransferase (U/l)	−0.08	0.497
Alkaline phosphatase (U/l)	0.087	0.457
Gamma-glutamyl transpeptidase (U/l)	−0.042	0.723
Prothrombin time (seconds)	0.224	0.053
Hemoglobin (g/dl)	−0.183	0.117
White blood cells (×10^3^/mm^3^)	0.102	0.385
Platelets (×10^3^/mm^3^)	−0.013	0.912
Grade of liver fibrosis	−0.06	0.607
Necroinflammatory activity	−0.107	0.362

BA: biliary atresia; IL-12p40: interleukin-12 p40 subunit; *r*: correlation coefficient.

## References

[1] Chardot C. (2006). Biliary atresia. *Orphanet Journal of Rare Diseases*.

[2] Mack C. L., Sokol R. J. (2005). Unraveling the pathogenesis and etiology of biliary atresia. *Pediatric Research*.

[3] Wildhaber B. E. (2012). Biliary atresia: 50 years after the first kasai. *ISRN Surgery*.

[4] Narayanaswamy B., Gonde C., Tredger J. M., Hussain M., Vergani D., Davenport M. (2007). Serial circulating markers of inflammation in biliary atresia—evolution of the post-operative inflammatory process. *Hepatology*.

[5] Wu J. F., Chiang B. L., Chen H. L., Lai H. S., Chang M. H., Ni Y. H. (2006). Impaired T-lymphocyte proliferation function in biliary atresia patients with chronic cholestatic jaundice after a Kasai operation. *Pediatric Research*.

[6] Ghoneim E. M., Sira M. M., Abd Elaziz A. M., Khalil F. O., Sultan M. M., Mahmoud A. B. (2011). Diagnostic value of hepatic intercellular adhesion molecule-1 expression in Egyptian infants with biliary atresia and other forms of neonatal cholestasis. *Hepatology Research*.

[7] Sira M. M., El-Guindi M. A., Saber M. A., Ehsan N. A., Rizk M. S. (2012). Differential hepatic expression of CD56 can discriminate biliary atresia from other neonatal cholestatic disorders. *European Journal of Gastroenterology & Hepatology*.

[8] Sira M. M., Sira A. M., Ehsan N. A., Mosbeh A. (2015). P-selectin (CD62P) expression in liver tissue of biliary atresia: a new perspective in etiopathogenesis. *Journal of Pediatric Gastroenterology and Nutrition*.

[9] Arafa R. S., Abdel Haie O. M., El-Azab D. S., Abdel-Rahman A. M., Sira M. M. (2016). Significant hepatic expression of IL-2 and IL-8 in biliary atresia compared with other neonatal cholestatic disorders. *Cytokine*.

[10] Li J., Bessho K., Shivakumar P. (2011). Th2 signals induce epithelial injury in mice and are compatible with the biliary atresia phenotype. *The Journal of Clinical Investigation*.

[11] Shivakumar P., Sabla G. E., Whitington P., Chougnet C. A., Bezerra J. A. (2009). Neonatal NK cells target the mouse duct epithelium via Nkg2d and drive tissue-specific injury in experimental biliary atresia. *The Journal of Clinical Investigation*.

[12] DeRusso P. A., Ye W., Shepherd R. (2007). Growth failure and outcomes in infants with biliary atresia: a report from the biliary atresia research consortium. *Hepatology*.

[13] El-Guindi M. A., Sira M. M., Sira A. M. (2014). Design and validation of a diagnostic score for biliary atresia. *Journal of Hepatology*.

[14] Russo P., Magee J. C., Boitnott J. (2011). Design and validation of the biliary atresia research consortium histologic assessment system for cholestasis in infancy. *Clinical Gastroenterology and Hepatology*.

[15] Davenport M., Tizzard S. A., Underhill J., Mieli-Vergani G., Portmann B., Hadžić N. (2006). The biliary atresia splenic malformation syndrome: a 28-year single-center retrospective study. *The Journal of Pediatrics*.

[16] Superina R., Magee J. C., Brandt M. L. (2011). The anatomic pattern of biliary atresia identified at time of Kasai hepatoportoenterostomy and early postoperative clearance of jaundice are significant predictors of transplant-free survival. *Annals of Surgery*.

[17] Marwan I., Soliman H., Saleh S. (2014). Outcome of 268 cases of biliary atresia: a single-center experience over seventeen years. *HPB*.

[18] Sangkhathat S., Patrapinyokul S., Tadtayathikom K., Osatakul S. (2003). Peri-operative factors predicting the outcome of hepatic porto-enterostomy in infants with biliary atresia. *Journal of the Medical Association of Thailand*.

[19] Wildhaber B. E., Coran A. G., Drongowski R. A. (2003). The Kasai portoenterostomy for biliary atresia: a review of a 27-year experience with 81 patients. *Journal of Pediatric Surgery*.

[20] Chung P. H. Y., Wong K. K. Y., Tam P. K. H. (2015). Predictors for failure after Kasai operation. *Journal of Pediatric Surgery*.

[21] Khanna V., Bhatnagar V., Agarwala S., Srinivas M., Das N., Singh M. (2016). Portal pressure and blood nitric oxide levels as predictors of outcome in biliary atresia. *Journal of Indian Association of Pediatric Surgeons*.

[22] Lee W.-S., Chai P.-F., Lim K.-S., Lim L.-H., Looi L.-M., Ramanujam T. M. (2009). Outcome of biliary atresia in Malaysia: a single-centre study. *Journal of Paediatrics and Child Health*.

[23] Sookpotarom P., Vejchapipat P., Chittmittrapap S., Chandrakamol B., Poovorawan Y. (2006). Short-term results of Kasai operation for biliary atresia: experience from one institution. *Asian Journal of Surgery*.

[24] El-Faramawy A. A., El-Shazly L. B., Abbass A. A., Ismail H. A. (2011). Serum IL-6 and IL-8 in infants with biliary atresia in comparison to intrahepatic cholestasis. *Tropical Gastroenterology*.

[25] Kobayashi H., Yamataka A., Lane G. J., Miyano T. (2002). Levels of circulating antiinflammatory cytokine interleukin-1 receptor antagonist and proinflammatory cytokines at different stages of biliary atresia. *Journal of Pediatric Surgery*.

[26] Mack C. L., Tucker R. M., Sokol R. J. (2004). Biliary atresia is associated with CD4+ Th1 cell-mediated portal tract inflammation. *Pediatric Research*.

[27] Yang Y., Liu Y. J., Tang S. T. (2013). Elevated Th17 cells accompanied by decreased regulatory T cells and cytokine environment in infants with biliary atresia. *Pediatric Surgery International*.

[28] Watford W. T., Hissong B. D., Bream J. H., Kanno Y., Muul L., O'Shea J. J. (2004). Signaling by IL-12 and IL-23 and the immunoregulatory roles of STAT4. *Immunological Reviews*.

[29] Oppmann B., Lesley R., Blom B. (2000). Novel p19 protein engages IL-12p40 to form a cytokine, IL-23, with biological activities similar as well as distinct from IL-12. *Immunity*.

[30] Sieburth D., Jabs E. W., Warrington J. A. (1992). Assignment of genes encoding a unique cytokine (IL12) composed of two unrelated subunits to chromosomes 3 and 5. *Genomics*.

[31] Trinchieri G. (1998). Interleukin-12: a cytokine at the interface of inflammation and immunity. *Advances in Immunology*.

[32] Klinke D. J. (2006). The ratio of P40 monomer to dimer is an important determinant of IL-12 bioactivity. *Journal of Theoretical Biology*.

[33] Holscher C., Atkinson R. A., Arendse B. (2001). A protective and agonistic function of IL-12p40 in mycobacterial infection. *Journal of Immunology*.

[34] Shimozato O., Ugai S., Chiyo M. (2006). The secreted form of the p40 subunit of interleukin (IL)-12 inhibits IL-23 functions and abrogates IL-23-mediated antitumour effects. *Immunology*.

[35] Wu J. F., Kao P. C., Chen H. L. (2012). A high serum interleukin-12p40 level prior to Kasai surgery predict a favourable outcome in children with biliary atresia. *Liver International*.

[36] Bessho K., Bezerra J. A. (2011). Biliary atresia: will blocking inflammation tame the disease?. *Annual Review of Medicine*.

[37] Mentink-Kane M. M., Cheever A. W., Wilson M. S. (2011). Accelerated and progressive and lethal liver fibrosis in mice that lack interleukin (IL)-10, IL-12p40, and IL-13Ralpha2. *Gastroenterology*.

[38] Huaux F., Arras M., Tomasi D. (2002). A profibrotic function of IL-12p40 in experimental pulmonary fibrosis. *Journal of Immunology*.

[39] Yao Y., Yang W., Yang Y. Q. (2014). Distinct from its canonical effects, deletion of IL-12p40 induces cholangitis and fibrosis in interleukin-2Ralpha(−/−) mice. *Journal of Autoimmunity*.

[40] Kalinski P., Vieira P. L., Schuitemaker J. H., de Jong E. C., Kapsenberg M. L. (2001). Prostaglandin E(2) is a selective inducer of interleukin-12 p40 (IL-12p40) production and an inhibitor of bioactive IL-12p70 heterodimer. *Blood*.

[41] Clarke A. W., Poulton L., Wai H. Y. (2010). A novel class of anti-IL-12p40 antibodies: potent neutralization via inhibition of IL-12-IL-12Rbeta2 and IL-23-IL-23R. *MAbs*.

